# How does the perceived green human resource management impact employee’s green innovative behavior? —From the perspective of theory of planned behavior

**DOI:** 10.3389/fpsyg.2022.1106494

**Published:** 2023-01-16

**Authors:** Dian Song, Yan Bai, Hainan Wu, Xiaoyuan Wang

**Affiliations:** ^1^School of Political and Public Administration, Soochow University, Suzhou, China; ^2^School of Finance and Public Administration, Anhui University of Finance and Economics, Bengbu, China; ^3^Business School, Soochow University, Suzhou, China

**Keywords:** theory of planned behavior, perceived green human resource management, green innovative behavior, system identity, green supply chain management

## Abstract

Employees’ green innovative behavior encouraged by enterprises plays an important role in the enterprise sustainable development. The study explores the impact of perceived green human resource management on employees’ innovative behavior. Drawing upon the planned behavior theory, this study examines how perceived green human resource management impact employees’ green innovation behavior. Through three-stage questionnaire survey, 207 samples are obtained and hierarchical regression is employed to test the hypothesis., Data analysis results show that perceived green human resource management has a directly positive effect on employees’ green innovative behavior. Green behavior intention, self-efficacy of environmental protection behavior, and identity with the company’s green environmental protection system are the mediators between perceived green human resource management and employees’ green innovative behavior. Meanwhile, the results demonstrate that there is a chain mediating relationship among these variables. In addition, green supply chain management moderates the relationship between the identity of a green environmental protection system and employees’ green innovative behavior. These conclusions transcend the macro perspective and open the black box between green human resource management and enterprise performance. Enterprise should take a holistic view to play the role of green human resource management and supply chain management in the implementation of environmental strategy.

## 1. Introduction

As the problem of environmental pollution becomes more and more serious, the public pays more and more attention to the environmental problems of enterprises. Faced with increasing environmental pressure, enterprises take measures to development the sustainable operation model from all around (Farrukh et al., 2022). Besides the enterprises’ environmental actions, employees’ green innovative behavior is the critical force that can help enterprise improve sustainability performance, produce less waste (Davis et al., 2020; Rongbin et al., 2022; Tu et al., 2022). Given that the employees’ innovative behavior is self-initiated and not prescribed by organization, enterprise need to identify the contextual and individual antecedents to arouse the employee’s motivation to act environmental (Davis et al., 2020; Rongbin et al., 2022; Tu et al., 2022). Green human resources management (GHRM) is one of the most critical measures that can motivates employee to conduct green innovative behavior (Tang et al., 2018; Napathorn, 2022). GHRM was developed by Wehrmeyer and Vickerstaff (1996) and become a hot research topic in recent years (Tang et al., 2018; Napathorn, 2022).

Many studies have revealed the impact of GHRM on employee’s green behavior and performance (Chaudhary, 2020; He et al., 2021; Aboramadan et al., 2022; Tuan, 2022; Ye et al., 2022). Dumont et al. (2017) pointed out that GHRM influences employees’ green behavior by constructing a green atmosphere, and personal green values moderates the relationship between a green atmosphere and employees’ green behavior. They find only a few scholars explored the relationship between GHRM and employee’s green innovative behavior. For example, taking GHRM as a mediator, scholars (Ahmad et al., 2021; Islam et al., 2021a,b) discuss supervisor’s ethical leadership style on subordinates’ green or pro-environmental work behavior. In the contemporary, to meet the sustainable development goal, employees’ environmental protection behavior is not enough for the enterprise’s sustainable development. It is imperative for the employee to conduct green innovative behavior, which is initiated by employees, not the enterprise (Li and Wu, 2017). Green innovative behavior plays a crucial role in continuously creating environmental benefits and improving the core competitiveness of enterprises under the pressure of multiple stakeholders (Zhou and Zhang, 2018; Hazarika and Zhang, 2019). Currently, a study (Odugbesan et al., 2022) found that green hard and soft talent management practices have significant influence on employees’ innovative work behavior. Scholars (Bhatti et al., 2022) pointed out that GHRM practices and the environmental innovative performance are positively correlated (Chaudhary, 2020; Aboramadan et al., 2022; Tuan, 2022; Ye et al., 2022). However, these studies adopt the macro perspective at the organizational level to elucidate the impact of formulated GHHM on employees’ innovative behavior, ignores the gap between the formulated GHRM and the perceived GHRM (Bowen and Ostroff, 2004; Luu, 2021). There is a gap between implementing and perceived HRM (Napathorn, 2022). Employee’s green innovative behavior is an individual level concept, while formulated GHRM is an organizational level concept. It is not suitable to directly examine the impact of organizational formulated GHRM on individual innovative green behavior with the use of OLS method. Therefor, it is necessary to examine perceived GHRM role in the HRM-performance relationship and captures the variations due to employee perceptions and interpretations (Bowen and Ostroff, 2004; Sanders and Yang, 2016). And it is necessary to adopt the employee-centric approach to analyze how the perceived GHRM practices drives the employee’s green innovative behavior (Paulet et al., 2021).

Meanwhile, researches have shown that organizational culture (Sathasivam et al., 2021), perceived environmentally-specific authentic leadership (Luu, 2021), national institutional and cultural contexts (Rajabpour et al., 2022), and effective communication moderate the relationship between GHRM and environmental sustainability performance. Especially, some scholars have pointed out that green supply chain management (GSCM), as a kind of environmental management strategy, affects the relationship between GHRM and performance (Longoni et al., 2018). Employees’ innovative green behavior will inevitably be affected by the company’s GSCM strategy. Nevertheless, the impact of GSCM on employees’ green innovative behavior is not fully investigated.

Therefore, this paper will address three problems to fill the above research gap: first, how does perceived GHRM promote the employee’s innovative behavior; second, what is the mediating mechanism of perceived GHRM on employees’ green innovative behavior; third, how does green supply chain management, as a core part of an enterprise’s green development strategy, moderate the relationship between perceived GHRM and employees’ innovative green behavior. Answers to these questions may contribute the literature in three ways. First, drawing on the planned behavior theory (PBT), we provide novel insights on the mechanism which can expound the impact of perceived GHRM on employees’ green innovative behavior. Second, we employ employee-centric approach to investigate the impact of GHRM on employee’s green innovative behavior, the analysis result will be more robust. And it is conducive to help redirect GHRM research paradigm from the organization level to individual level in line with the HRM research paradigm (Sanders and Yang, 2016; Paulet et al., 2021). Third, we explore the moderation effect of GSCM, which is helpful to deepen the understanding of the situational factors that affect employees’ green innovation behavior. This paper is organized as follows: first, introduction section to provide the researching background; second, literature review and reasoning logistic for our hypothesis; third, the methods of the study; fourth, the analysis and results; the last, the discussion and conclusion.

## 2. Theoretical background and hypothesis

### 2.1. Theoretical background

The theory of planned behavior (TPB) originated from the theory of reasoned action (TRA) proposed by Ajzen and Fishbein in 1975. TRA holds that behavioral intention (BI) is the direct factor in determining behavior (Yang et al., 2012), and is influenced by behavioral attitude (BA), subjective norms (SN), and perceived behavioral control (PBC) (Trafimow et al., 2002). BA refers to an individual’s assessment of how much one likes or dislikes performing a particular behavior, which is usually the most powerful predictor variable of BI. Factors influencing an individual’s BA can be divided into endogenous and exogenous attitudes. The former arises from internal traits of individuals, while the latter comes from external stimuli including employee identification and attitudinal disposition in this study. SN refers to the social pressure when individuals consider adopting a particular behavior. Reno et al. (1993) classify subjective norms as injunctive norms, regulating what others think individuals should do, descriptive norms, about their behaviors of themselves, and personal norms or moral norms, regarding what individuals believe they should do. PBC refers to the ease or difficulty with which an individual believes he or she can control and perform a behavior, such as an employee self-efficacy (Hagger and Chatzisarantis, 2005). It relies on both internal control, which is derived from Bandura’s self-efficacy theory and external control which is about the facilitation or inhibition of behavior by other factors such as the level of cooperation from colleagues, resources, or time constraints perceived by the individual (Kraft et al., 2005).

### 2.2. Hypotheses

#### 2.2.1. Green HRM and employees’ green innovation behavior

GHRM incorporates environmental norms into human resource activities (Renwick et al., 2013; Dumont et al., 2017; Amrutha and Geetha, 2020). It is an environment-focused HRM system, whose aim is to increase employees’ awareness, knowledge, skills, and motivation in enterprise’s environmental sustainable development (Ren et al., 2018). Green human resource management is a bundle of HRM practices, which combines green management practices and HRM processes, including recruitment and selection, training and development, compensation and benefits, performance management, and employee engagement (Zibarras and Coan, 2015; Ren et al., 2018; Tang et al., 2018). GHRM encourages employees to carry out green behaviors at work (Kim et al., 2019). However, the designed GHRM by the enterprise will not be fully implemented and will be perceived variously by employee due to individuals’ personality, attribution style or value (Batt and Hermans, 2012; Sanders and Yang, 2016). Perceived GHRM refers to the perceived GHRM by the employee, regardless of proactive or reactive. It is not the formulated HRM by the enterprise. While an enterprise may design a variety of HRM practices, they are not perceived by the employee for many reasons. These practices will not influence employees. Following this logic, only the perceived GHRM can influence employees (Renwick et al., 2013; Paillé et al., 2014; Ren et al., 2018; Lu et al., 2022) Perceived GHRM is significant predictor of employee behavior (Yusliza et al., 2021).

Employees’ green innovative behavior refers to individuals’ behaviors in the everyday works, including manufacturing new products or providing service (Rongbin et al., 2022). It involves green and novel idea generation, promotion and utilization (Li et al., 2019; Li Y.-B. et al., 2020; Singh et al., 2020). Employees’s green innovative behavior has two distinguishing characteristics: proactive and prosocial. The former highlights that it is nonmandatory, discretionary, and self-directed initiative (Dumont et al., 2017; Robertson and Carleton, 2017; Tian and Robertson, 2019; Rubel et al., 2021; Biswas et al., 2022; Munawar et al., 2022). The influence of perceived GHRM on employees’ green innovative behavior can be examined with the use of TPB from the perspective of HR practices. The perceived green recruitment and selection practices will make environmental tendencies an important factor in employee promotion, which will boost employees’ intention to act environment-friendly. The perceived green training practices will help the employee to form green values and develop the ability to implement green innovative behavior. As a form of subjective norms, it will promote an employee to carry out green innovative behavior with high consciousness and innovative awareness, and is conducive for the employee to develop innovative competency. The perceived green performance management and compensation practices highlight that if employees act with a high characteristic of green innovative behavior, the enterprise will reward them with high-level pay. It will enhance employees’ motivation of implementing green innovative behavior. The perceived empowerment and team practices will enable individuals to feel a supportive atmosphere in doing green innovative behavior from others, which is a kind of the subjective norm. Therefore, we propose the following hypothesis.

*H1*: Perceived GHRM positively relates to Employees' Green Innovation Behavior.

#### 2.2.2. The mediating effect of intention, self-efficacy, and identity

TPB proposed that individuals’ behavior is affected by behavioral intention (BI), which in turn is the combined result of variables, such as personal behavior attitude (BA) and perceived behavioral control (PBC) (Armitage and Conner, 2001). Research shows that the green behavior intention will be influenced by green organization identity (Chen, 2011). Green organization identity refers to the individual’s interpretive scheme on organization’s environmental management and protection system which will impact the individual’s behavior. Green organization identity is embodied in employees’ identification with the green environmental protection system (IWTGPS), which reflects the employees’ recognition of the enterprise’s green strategy including its necessity, and effectiveness. Studies have shown green organization identity impacts individual’s organizational citizenship behavior for the environment (Liu et al., 2021), sustainability exploration innovation (SER) (Xing et al., 2019), green innovation performance (Chang and Chen, 2013) and green creativity (Song and Yu, 2018). IWTGPS can promote employees to establish environmental awareness and green management, and behavior (Chang and Chen, 2013; Xing et al., 2019). Therefor, employees, with a high sense of identity with the enterprise’s green environmental protection system, will have a high likelihood to conduct green innovative behavior from the view of TPB. (Gioia and Thomas, 1996; Chen, 2011; Chang and Chen, 2013; Song and Yu, 2018; Xing et al., 2019; Liu et al., 2021).

Meanwhile, as a type of BI, an employee’s IWTGPS will be affected by the employee’s green environmental protection intention (BA) and environmental behavior self-efficacy (PBC) in the light of TPB. Green self-efficacy refers to the employees’ belief about his competencies to engage and accomplish environment-related tasks (Chen et al., 2015; Faraz et al., 2021). Green self-efficacy affects employee’s green behavior (Adnan, 2021), green creativity (Chen et al., 2015), and pro-environmental behavior (Faraz et al., 2021). Employees with high self-efficacy will exert more resource, time and commitment to works and tolerate failure (Bandura, 1997; Zhang et al., 2022) Thus, we propose that an employee’s environmental protection intention and environmental behavior self-efficacy are positively related to his green innovative behavior, and the relationship will be mediated by IWTGPS. That is, only when individuals have the will for green innovation, they will continue to strengthen their willingness in the action, till the final green innovation behavior gets implemented.

Furthermore, on one hand, perceived GHRM by employees can strengthen their attitude toward green environmental protection behavior and felt responsibility by conveying the organization’s concern for corporate ES strategy and social responsibility. Which is consistent with the company’s entire green environmental protection strategy (Lu et al., 2022). At the same time, perceived GHRM can enhance employee’s organizational identification, which in turn leads to green behaviors (Chaudhary, 2020). On the other hand, perceived GHRM can help the employee develop conscious awareness and innovation ability when implementing environmental protection behaviors, and pave the way for employees to recognize the organizational green environmental protection system from the perspective of ability self-control and broadening (Zhou and Zhang, 2018). The generation of green environmental protection intention and the strengthening of self-efficacy of environmental protection behavior will be affected by perceived GHRM (Cherian and Jacob, 2012; Gill, 2012; Tang and Sun, 2021). In combination with H1, we propose the following hypothesis:

*H2a*: Green environmental protection intention and green system identity are the chain mediators between perceived GHRM and employees’ green innovative behavior.

*H2b*: Environmental behavior self-efficacy and green system identity are the chain mediators between perceived GHRM and employees’ green innovative behavior.

#### 2.2.3. The moderating role of green supply chain management

GSCM refers to the actions to reduce consumption of raw resources, waste in internal operational processes, and increase the use of recycled/recyclable materials in external operational processes (Sarkis, 2012; Gimenez and Sierra, 2013). GSCM reflects the enterprise’s environmental awareness in the process of product development, purchasing, distribution, and reverse logistics (Chan et al., 2016). It is a kind of environmental strategy. (Chan et al., 2016; Li G. et al., 2020). Some researches show that GSCM mediate the relationship between GHRM and performance (Longoni et al., 2018). In contrast, some scholars found GHRM influence the implementation of GSCM process greatly (Kumar et al., 2019).

**Figure 1:**
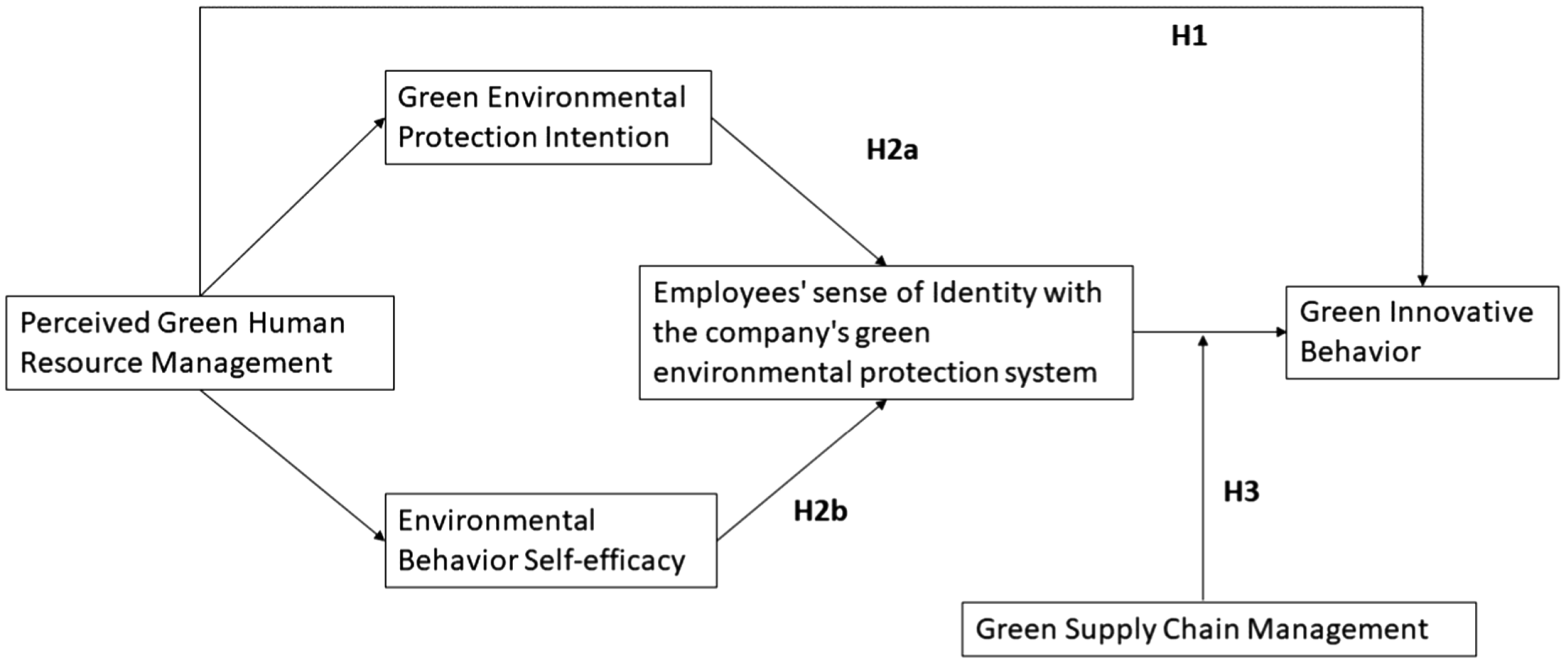
Research framework.

Green supply chain management is a modern management mode that comprehensively considers the environmental impact and resource efficiency in the whole supply chain (Zheng and Xie, 2017) As a complex system to improve economic and environmental benefits, the green supply chain carries out unified organizational planning and coordinated management, which consists of environmentally purchasing materials, energy-saving design, reverse logistics, internal environmental management, cooperation with downstream buyers, and recycling As supply chain management involves various departments and jobs, it has become a research hotspot. In carrying out GSCM model, enterprise will train employees, acquire ISO 14001 certification, strengthen waste disposal (Li G. et al., 2020). GSCM is an effective tool for environmental performance improvement (Chan et al., 2016; Li G. et al., 2020). Therefore, green supply chain management can strengthen employees’ sense of identity with corporate environmental protection strategies, and ultimately promotes green innovative behaviors. It will strengthen the relationship between the perceived GHRM and employees’ green innovative behavior. Thus, we propose the following hypothesis (Figure 1).

*H3*: Green supply chain management positively moderates the relationship between green environmental protection identity and employees’ green innovative behavior, that is, the higher the level of green supply chain management, the stronger the relationship between identity and green innovation behavior, and vice versa.

Based on the above assumptions, the analysis framework is as follows.

Both environmental protection intention and environmental behavior self-efficacy are the chain mediators between perceived GHRM and green innovative behavior through producing employee’s sense of identify with company’s green environmental protection system. And in this process, GSCM plays a moderating role, that is, the higher the level of GSCM, the stronger the relationship between employee’s identity and green innovation behavior, and vice versa.

## 3. Methodology

### 3.1. Samples

In this study, data were collected by questionnaire. The samples are mainly from Suzhou. We use on-site and online distribution methods to survey and distributed 260 questionnaires, including 214 paper questionnaires and 46 electronic questionnaires. After excluding 53 invalid questionnaires, 207 valid questionnaires were returned, which accounted for 79.62%. Respondents are from chemical, manufactory, pharmaceutical, and hotel sectors. Their jobs are mainly production, supply chain managers, technical workers, R&D and others. Under their consent, paper-pencil or online questionnaire was distributed. The data collection was organized in three stages. During stage 1, employees answered questions about perceived GHRM, green supply chain management, and demographics. During stage 2, about 1 month later, employees answered the questions about mediators, such as green environmental protection intention, green system identity, environmental behavior self-efficacy, and green system identity. During stage 3, about 1 month after stage 2, the employee answered the questions about green innovative behavior. Sample profiles are shown in Table 1.

**Table 1:** Survey samples.

Variable	Category	Numbers	Percentage (%)
Gender	Man	98	47.34%
	Woman	109	52.66%
Age	Less than 25	15	7.25%
	26–35	160	77.29%
	36–45	31	14.98%
	More than 46	1	0.48%
Enterprise size (Number of people)	More than 3,000	44	21.26%
	300–2,999	67	32.37%
	101–299	48	23.19%
	Less than 100	48	23.19%
Working time(year)	Less than 4	34	16.43%
	5–9	88	42.51%
	10–15	57	27.54%
	16–20	18	8.70%
21 or above	10	4.83%

### 3.2. Measures

The Likert-5 scale was used, ranging from 1 (“strongly disagree”) to 5 (“strongly agree”). Perceived GHRM is adopted from Sun et al. (2007), including 6 items. The intention of green environmental protection involves 4 items and the self-efficacy of environmental behavior includes 3 items adopted from Cordano and Frieze (2002). The measurement of the green innovation behavior refers to the method of Ng and Lucianetti (2016), which contains 5 items. Employees’ identification with the enterprise’s green environmental protection system uses items from Mael and Ashforth (1992), including 3 items. Green supply chain management is adopted from the research of Jabbour and Jabbour (2016), with 5 items.

### 3.3. Reliability and validity

We use confirmatory factor analysis (CFA) to calculate the reliability and validity. The results are summed in Table 2.

**Table 2:** The results of confirmatory factor analysis.

	RMSEA	SRMR	CFI	TLI	*X* ^2^	DF	C.R.	AVE
Six-factor model	0.070	0.048	0.909	0.895	529.869	283	0.961	0.608
Five-factor model	0.111	0.093	0.795	0.769	1030.306	289	0.957	0.565
Three-factor model	0.135	0.105	0.691	0.661	1413.917	296	0.939	0.465
One-factor model	0.161	0.118	0.574	0.535	1753.193	275	0.940	0.385

1. The six-factor model means that all variables are listed separately. 2. The five-factor model combines green environmental protection intention and environmental protection behavior self-efficacy into one variable. 3. The three-factor model combines green human resource management, green environmental protection intention, environmental protection behavior self-efficacy, and employees. The identification with the corporate green environmental protection system is combined into one variable. 4. The single-factor model combines all variables into one variable.

From Table 2, we can see the goodness of fit of the six-factor model is good, as follows: X2/DF = 1.872 < 3, RMSEA = 0.070 < 0.080, SRMR = 0.048, CFI = 0.909, TLI = 0.895. C.R. = 0.961, AVE = 0.608. It is significantly better than five-factor, three-factor, and single-factor models (see Table 2). Each variable’s reliability and validity value is as follows: perceived GHRM (*α* = 0.875, AVE = 0.660, CR = 0.921), Green environmental protection intention (*α* = 0.845, AVE = 0.713, CR = 0.909), environmental behavior self-efficacy (*α* = 0.848, AVE = 0.788, CR = 0.918), green innovation behavior (*α* = 0.865, AVE = 0.687, CR = 0.916), employees’ sense of identity with enterprise’s green environmental protection system (*α* = 0.709, AVE = 0.638, CR = 0.840) and green supply chain management (*α* = 0.880, AVE = 0.710, CR = 0.924). These results show that the reliability and validity of the questionnaire have reached an acceptable level.

Furthermore, we use Harmon’s method to test the common method bias. All items are loaded into a latent variable. The results (RMSEA = 0.161, SRMR = 0.118, CFI = 0.574, TLI = 0.535, *X*^2^ = 1753.193, DF = 275, *α* = 0.939, CR = 0.940, AVE = 0.385) indicates that the common method bias is not a serious problem.

### 3.4. Correlation coefficient

The analysis of the correlation coefficient of each variable is shown in Table 3.

**Table 3:** Mean, standard deviation, and correlation coefficient.

Variable	Mean	Std	1	2	3	4	5	6	7	8
1.Gender	1.768	0.423	1.000							
2.Age	2.087	0.485	0.075	1.000						
3. PGHRM	3.122	0.732	0.116	0.098	1.000					
4. EI	3.783	0.593	−0.091	0.049	0.338^**^	1.000				
5. SE	3.416	0.688	−0.079	0.110	0.522^**^	0.485^**^	1.000			
6 GIB	3.434	0.609	0.095	0.181^**^	0.558^**^	0.486^**^	0.686^**^	1.000		
7. IN	3.759	0.503	0.100	0.146^*^	0.333^**^	0.541^**^	0.432^**^	0.548^**^	1.000	
8.GSCM	3.557	0.130	−0.102	0.130	0.514^**^	0.325^**^	0.408^**^	0.584^**^	0.537^**^	1.00

**p < 0.05, *p < 0.10; PGHRM, Perceived Green Human Resource Management; EI, Green Environmental Protection Intention; SE, Environmental Behavior Self-efficacy; GIB, Green Innovation Behavior; IN, Employees’ Sense of Identity with the company’s green environmental protection system; GSCM, Green Supply Chain Management.

The data preliminarily verify the hypothesis. It is shown that green innovative behavior is positively correlated with perceived green HRM, green environmental protection intention, environmental protection behavior self-efficacy, employees’ recognition of the enterprise’s green environmental protection system, and green supply chain management.

## 4. Data analysis results

### 4.1. The relationship between perceived GHRM and employees’ green innovation behavior

This study uses SPSS regression analysis to test the hypotheses, and the results are shown in Table 4.

**Table 4:** Regression analysis results.

Variable	GIB Model1	EI Model2	SE Model3	IN Model4	GIB Model5	GIB Model6
Intercept	1.637^***^	3.161^***^	1.913^***^	1.275^***^	0.675^**^	2.152^***^
Gender	0.022	−0.187	−0.098^*^	0.180^***^	0.049	−0.058
Age	0.126^**^	0.131^**^	0.067	0.092	0.127^***^	0.144^**^
PGHRM	0.543^***^	0.416^***^	0.532^***^			
EI				0.377^***^		
SE				0.485^***^		
Intention					0.641^***^	0.384^***^
GSCM						0.437^***^
I*GSCM						0.129^**^
*F*	32.995^***^	10.296^***^	17.564^***^	28.755^***^	30.646^***^	33.454^***^
*R* ^2^	0.328	0.132	0.297	0.363	0.312	0.454
Adj *R*^2^	0.318	0.119	0.287	0.350	0.302	0.442

^***^p < 0.01, ^**^p < 0.05, ^*^p < 0.10; PGHRM, Perceived Green Human Resource Management; EI, Green Environmental Protection Intention; SE, Environmental Behavior Self-efficacy; GIB, Green Innovation Behavior; IN, Employees’ Sense of Identity with the company’s green environmental protection system; GSCM, Green Supply Chain Management.

It can be seen from Model 1 that the regression coefficient of perceived GHRM on employees’ green innovative behavior is 0.543 (*p* < 0.01), and H1 is supported. From model 2 and model 3, it can be seen that the coefficients of perceived GHRM on EI and SE are 0.416 (*p* < 0.01) and 0.532 (*p* < 0.01) respectively. From Model 4, we can see the coefficients of EI and SE on employees’ intention 0.377 (*p* < 0.01) and 0.485 (*p* < 0.01) respectively. Model 5 shows the coefficient of intention on green innovative behavior is 0.641 (*p* < 0.01). It provides a preliminary test for H2.

### 4.2. The mediating role between perceived green HRM and employees’ green innovative behavior

This study analyzes the mediating role of employees’ identification with the enterprise’s green environmental protection system on employees’ green environmental protection intention, environmental protection behavior self-efficacy, and employees’ green innovation behavior. Employing Hayes’s PROCESS program, we explore the two chain mediating paths to test H2. Data analysis results shows that the chain mediating effect value of the former is 0.136 [0.072, 0.226], while the latter is 0.272 [0.185, 0.375]. This proves that the above two chain mediation paths are both valid, and the mediating effect of the latter is higher than that of the former. H2 is supported.

### 4.3. The moderating role of green supply chain management

**Figure 2:**
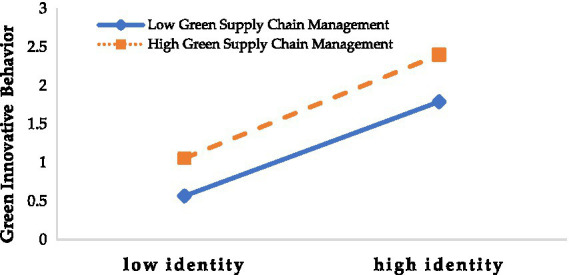
The moderating role of green supply chain management.

From model 6, we can see the interaction coefficients of green supply chain management and intention on employee’s green innovative behavior is 0.129, hypothesis H3 is verified. Further, we divide samples into two subgroups based on green supply chain management. The results show that when one standard deviation is subtracted, *β* is 0.358, and the confidence level is 95%. The interval is between 0.173 and 0.543; when one standard deviation is added, *β* is 0.411, and the confidence interval at the 95% level is between 0.234 and 0.588. From this, we can see that both results do not include 0 points, as shown in Figure 2. Therefore, the significance of the moderating effect of green supply chain management has been further verified, the interaction effects are as in Figure 2.

## 5. Conclusion and implications

### 5.1. Conclusion

Based on the above analysis, the findings of this study are as follows. First, perceived GHRM has a significant positive impact on employees’ green environmental intentions and environmental behavior self-efficacy. Green environmental intentions and environmental behavior self-efficacy also significantly and positively affect employees’ green innovative behavior. Second, employees’ identification with the company’s green environmental protection system plays a significant mediating role in the transformation of green environmental protection intentions and environmental behavior self-efficacy into green innovation behaviors. The two intermediary influence paths (i.e., perceived GHRM - green environmental protection intention - employee’s identity with the enterprise’s green environmental protection system - green innovation behavior, and perceived GHRM - environmental protection behavior self-efficacy - employee’s identity with the enterprise’s green environmental protection system) are confirmed. Third, green supply chain management has a positive moderating effect on the mechanism of employee’s identity with the enterprise’s green environmental protection system influencing employees’ green innovative behavior.

### 5.2. Theoretical and practical implications

Theoretical implication is threefold. First, previous research adopted the macro perspective to examine the impact of GHRM on green behavior (Rongbin et al., 2022; Zhang et al., 2022). However, a few studies adopted the micro perspective to analyze the influence. Our research applies TPB to examine the impact of perceived GHRM on employees’ innovative behavior which is more critical for enterprise sustainable than green behavior at the individual level. Our study shows that perceived GHRM affects the employees’ identity with the company’s green environmental protection system via employees’ green environmental protection intention and environmental protection behavior self-efficacy. The results precisely clarify the chain mediation linkage between perceived GHRM and employees’ green innovation behavior and deepen our understand of the black box between GHRM and enterprise environmental performance. Second, scholars (Sanders and Yang, 2016) highlight that the process of HRM may be more crucial than the content of HRM. The perceived GHRM is a paradigm of process HRM. Therefore, the conclusions of this study prove the core views and the validity of process HRM. Third, previous studies (Nejati et al., 2017; Saeed et al., 2022) have explored the relationship between GHRM and GSCM, and many of them argued that GHRM is the driver of GSCM. Nevertheless, our research show that GSCM, as a kind of enterprise’s environmental strategy, moderates the relationship between GHRM and employees’ innovative behavior. The contingency theory of strategic HRM points out that HRM can play a better role only when it matches with other management practices. Further on this basis, our research reveals that the interactions GHRM and GSCM impact the employees’ green innovative behavior and deepen the understanding of the role of supply chain management in the contingency theory of strategic human resource management.

Practical implications are as follows. First, HR department should guide employees consciously *to l*earn about green behaviors through professional training and green knowledge sharing (Islam et al., 2021a; Ahmed et al., 2022), thereby enhancing their psychological sense of self-control for green innovation behaviors, so as to independently carry out green innovation behaviors. Second, enterprise ought to set up a position dedicated to the construction of environmental protection culture, responsible for coordinating the construction of corporate green culture, to push the company’s green environmental protection culture closer to the employee, and to arouse individual resonance to integrate into his work. Third, enterprise should take a holistic view to play the role of GHRM and GSCM. Enterprise need to keep the match between GHRM and GSCM, try to utilize information and communication technology in GSCM (Batool et al., 2019), and train employees in the green procurement, production and innovation in the process of GSCM.

### 5.3. Limitations and future research

While the study tests the hypothesis, there also are some limitations. First, we collect GSCM data from the employee, not from managers. It may lead to measurement bias. In the future, we can collect data from multisource to conduct an integrated macro and micro level, and provide a comprehensive framework to discuss the interaction effects of perceived GHRM and GSCM. Second, perceived GHRM originates the paradigm of process HRM, which stresses that the strength as well as attribution style is critical in the prediction of employees’ behavior. These variables are not incorporated in the study. It provides another future research direction.

## Data availability statement

The original contributions presented in the study are included in the article/supplementary material, further inquiries can be directed to the corresponding author.

## Author contributions

DS: conceptual work and literature review. HW: writing, translating, editing, and revise. YB: writing, translating, and editing. XW: editing and revise. All authors contributed to the article and approved the submitted version.

## Conflict of interest

The authors declare that the research was conducted in the absence of any commercial or financial relationships that could be construed as a potential conflict of interest.

## Publisher’s note

All claims expressed in this article are solely those of the authors and do not necessarily represent those of their affiliated organizations, or those of the publisher, the editors and the reviewers. Any product that may be evaluated in this article, or claim that may be made by its manufacturer, is not guaranteed or endorsed by the publisher.
